# Domain Diversity and Polarization Switching in Amino Acid β-Glycine

**DOI:** 10.3390/ma12081223

**Published:** 2019-04-15

**Authors:** Daria Vasileva, Semen Vasilev, Andrei L. Kholkin, Vladimir Ya. Shur

**Affiliations:** 1 School of Natural Sciences and Mathematics, Ural Federal University, Ekaterinburg 620000, Russia; daria.vasileva@urfu.ru (D.V.); vasilev.semen@gmail.com (S.V.); vladimir.shur@urfu.ru (V.Y.S.); 2 Department of Chemical Science, Bernal Institute, University of Limerick, V94 T9PX Limerick, Ireland; 3 Department of Physics & CICECO-Aveiro Institute of Materials, University of Aveiro, Aveiro 3810-193, Portugal

**Keywords:** organic ferroelectrics, amino acids, domain structure, nonpolar cut, local polarization reversal, PFM, glycine

## Abstract

Piezoelectric materials based on lead zirconate titanate are widely used in sensors and actuators. However, their application is limited because of high processing temperature, brittleness, lack of conformal deposition and, more importantly, intrinsic incompatibility with biological environments. Recent studies on bioorganic piezoelectrics have demonstrated their potential in these applications, essentially due to using the same building blocks as those used by nature. In this work, we used piezoresponse force microscopy (PFM) to study the domain structures and polarization reversal in the smallest amino acid glycine, which recently attracted a lot of attention due to its strong shear piezoelectric activity. In this uniaxial ferroelectric, a diverse domain structure that includes both 180° and charged domain walls was observed, as well as domain wall kinks related to peculiar growth and crystallographic structure of this material. Local polarization switching was studied by applying a bias voltage to the PFM tip, and the possibility to control the resulting domain structure was demonstrated. This study has shown that the as-grown domain structure and changes in the electric field in glycine are qualitatively similar to those found in the uniaxial inorganic ferroelectrics.

## 1. Introduction

Currently, inorganic ferroelectrics such as lead zirconate titanate (PZT), lithium niobate (LNO) and barium titanate (BTO) are widely used as efficient piezoelectrics, pyroelectrics, memory cells, and electrooptic modulators [1]. They possess high switchable polarization, strong piezoelectric response, and remarkable pyroelectric and electrooptic properties, but have a number of disadvantages that preclude their use in biomedical applications [2,3]. First of all, they are not biologically compatible, and they require encapsulation for contact with biological environments. Secondly, their processing requires high temperatures, so their miniaturization and integration with microelectromechanical systems (MEMS) is difficult. Moreover, inorganic materials are brittle and do not allow conformal deposition. Novel organic or polymer materials, e.g., polyvinylidene fluoride (PVDF), do not seem to have these disadvantages, but still may require protection from the tissue or cell when in direct contact [4]. Such materials should be nontoxic, noninjurious, and biocompatible, because they are involved in tissue engineering [5], minimally invasive sensors [6], drug delivery [7], etc. During the last decade, there have been several reviews describing the possible applications of bioorganic [8] and inorganic–organic materials [9].

In this context, piezoelectric or pyroelectric materials made from the same building blocks as those used by nature (e.g., amino acids or peptides) may be used, e.g., for bone repair or axonal regeneration after nerve injury. Until recently, biomolecular piezoelectrics had properties which were by far inferior to their inorganic counterparts, thus making their applications unfeasible. Recent studies have revealed that the softness of hydrogen bonds in some representative classes of biomolecular polar materials may be the origin of strong piezoelectric and pyroelectric effects at room temperature. For example, piezoelectric coefficients in self-assembled short peptides (diphenylalanine (FF)) are practically the same as in the popular transducer material LNO [10].

Early studies on amino acids have demonstrated that many of them are non-centrosymmetric, though piezoelectric coefficients were found to be quite low [11]. However, recent investigations have shown that the peculiar molecular packing in amino acids may be accountable for the strong piezoelectric response, as exemplified by glycine [12]. Glycine is the simplest amino acid and is polymorphic in nature [13,14] and ferroelectric in its polar β-phase [15]. Recent modeling results have shown that glycine has a very high spontaneous polarization (~70 μC/cm^2^ [16]), which is comparable to best inorganic materials such as LNO (~78 μC/cm^2^) [17] and BiFeO_3_ (~95 μC/cm^2^) [18]. Glycine serves as being the symbolic origin of life when found in the meteorites and comets. It is a well-known drug useful for treating schizophrenia, stroke, benign prostatic hyperplasia (BPH), and some rare inherited metabolic disorders. It is also used to protect kidneys from the harmful side effects of certain drugs used after organ transplantation, as well as to protect the liver from the harmful effects of alcohol. Its strong piezoelectricity and ability to switch polarization under the action of electric field adds new functionality that can be further used in biomedical applications. The high nonlinear optical coefficients previously observed in glycine [19] allow for the creation of periodical domain structures widely used for light frequency conversions. As β-glycine microcrystals are typically grown on a substrate by evaporation from solution [20], only nonpolar surfaces are available for the study of domain structure. As such, it is only possible to investigate the polarization reversal and evolution of the domain structure on a nonpolar cut. Such a possibility has been recently demonstrated both theoretically [21] and experimentally [22,23]. It should be noted that piezoelectric response is typically governed by both intrinsic and extrinsic effects related to domain wall motion [24]. Therefore, the study of micro- and nanodomain structures in glycine is also important from this point of view.

## 2. Materials and Methods

In this work, glycine crystals of micron sizes (typical sizes of 50 × 500 × 20 µm^3^) that were grown from the drying drop of an aqueous solution deposited on a substrate were investigated. The polar axis of the investigated crystals lies in the plane of the substrate. To prepare the solution, glycine powder (≥99%), purchased from Sigma-Aldrich (Belgium), was dissolved in deionized water produced using the Elix 10 ultrapure water preparation system (Millipore, France). To grow glycine crystals from a drop, solutions of glycine powder in deionized water with a concentration of 10 mg/mL were used. A drop of 50 μL solution was dried on a substrate at room temperature and about 25% humidity. Platinum-coated Si/SiO_2_ substrates (Inostek, Ansan, South Korea) were used. The identification of polymorphic phases was carried out using the Alpha 300 AR+ Raman microscopy system (WiTec GmbH, Ulm, Germany). It should be noted that the crystals grew in ferroelectric phase, so that the domain structure was partly determined by the growth defects.

In order to determine the direction of the crystal axes in all three polymorphic phases of glycine, Raman spectra with polarization of laser radiation directed along two selected directions in a crystal were measured [25]. Imaging of the topography and domain configurations of glycine crystals with high spatial resolution was carried out in contact mode using piezoresponse force microscopy (PFM) implemented in the NTEGRA Prima nanolaboratory (NT-MDT SI, Zelenograd, Russia) with an external lock-in amplifier SR-830A (Stanford Research, Stanford, CA, USA) and SPM Asylum MFP-3D (Asylum Research, Oxford Instruments, Santa Barbara, CA, USA). We used conductive cantilevers coated in Pt with a force constant of 2–5 N/m and a resonant frequency of 60–150 kHz. During scanning and measurements, an alternating electrical voltage with an amplitude of up to 40 V and a frequency of 20 kHz was applied to the probe, and the substrate was grounded. The maximum applied voltage was 200 V, and the frequency ranged from 1 Hz to 2 MHz. The special sample holder allowed the measurements to be carried out in temperatures ranging from 20 °C to 120 °C.

Local polarization reversal was performed using single rectangular pulses with an amplitude from 50 Vto 100 V, and duration ranged from 15 s to 60 s. Two switching modes were used: (i) With tip withdrawal after the end of each pulse and (ii) without withdrawal of the probe, so that the tip remained in contact with the surface after switching.

## 3. Results and Discussion

β-glycine is a uniaxial ferroelectric as it belongs to the monoclinic system (P2_1_) and has a single crystallographic polar axis along which the vector of spontaneous polarization can be directed. The polar axes of the investigated crystals were directed along the long side of the crystals and lay in the plane of the substrate. The shape and orientation of β-glycine crystals grown on conductive substrates made it only possible to investigate the domain structure and its evolution during polarization reversal on a nonpolar cut [21,24,26,27] as the polarization is always directed along the longest crystal side.

### 3.1. As-Grown Domain Structure

**Figure 1:**
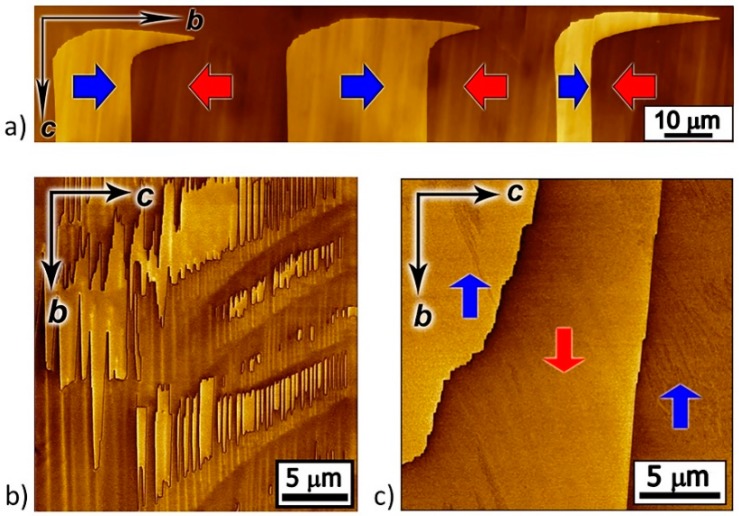
Piezoresponse force microscopy (PFM) images of as-grown domain structures. Lateral PFM contrast on nonpolar surfaces of β-glycine representing three types of as-grown domain structures: (**a**) Strip-like domains with flat charged domain walls, (**b**) quasiperiodic ensembles of needle-like domains, and (**c**) large domains with irregular-shaped domain walls. Red and blue arrows indicate the direction of spontaneous polarization. Arrows b and c show the directions of crystal axis.

In the as-grown β-glycine crystals, three types of domain configurations were typically observed: (i) Strip-like domains with flat charged domain walls (Figure 1a), (ii) quasiperiodic ensembles of needle-like domains of submicron width (Figure 1b), and (iii) large domains with irregular-shaped domain walls (Figure 1c).

**Figure 2:**
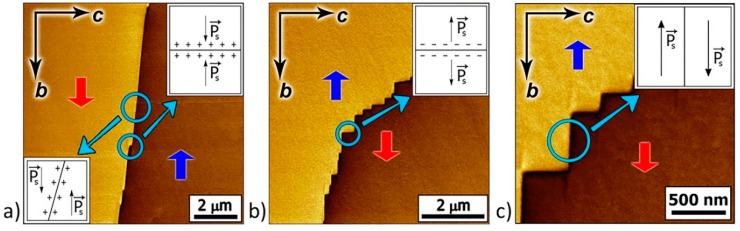
PFM images of the domain walls. Three types of the domain wall fragments: Charged (**a**) head-to-head, (**b**) tail-to-tail, and (**c**) neutral. Lateral PFM contrast. Red and blue arrows indicate the direction of spontaneous polarization within domains. Arrows b and c show the directions of crystal axis.

The domain walls on nonpolar cut consisted of charged and neutral fragments. The head-to-head and tail-to-tail charged fragments (Figure 2a,b) with a bound charge density σ = 2P_s_ were oriented along c axis, while the uncharged neutral fragments were oriented along the polar axis b (Figure 2c). We also found slightly inclined domain walls with an angle to the polar axis of about 6°, which are also charged, but the density of the bound charge was substantially less (σ = 2P_s_·sinα).

Most tail-to-tail type charged domain walls were perpendicular to the polar axis, while head-to-head type walls were more often inclined. This suggests that the charged tail-to-tail domain walls are conductive and facilitate and accelerate the bulk screening of the depolarizing fields created by the bound charges [28,29,30]. On nonconductive head-to-head-type charged domain walls, the depolarizing fields were not fully compensated, which led to a change of the domain wall shape to reduce the density of bound charges.

The formation of the strip-like as-grown domain structure with flat charged domain walls and their smooth change in orientation near the edges of the crystal can be attributed to the growth mode of glycine crystals. We suppose that the fast growth facet was oriented perpendicular to the polar axis and consisted of the growth sectors separated by boundaries [31]. These boundaries are internal surfaces over which the edges between neighbored faces have been swept during growth. These interfaces are readily seen in the topography images (not shown) and may contain an increased concentration of defects or deviations from the stoichiometric composition [32,33]. Nonuniform concentration of defects at different stages of crystal growth can occur because of fluctuations in the growth rates of the faces, which are caused by cyclic changes of the temperature and oversaturation [34,35]. The local direction of polarization is determined by the defect concentration gradient; therefore, the domain walls were localized at the places of the gradient sign changes [33].

The formation of two other types of the as-grown domain structure can be attributed to the polarization switching in the strip-like domains under the action of the pyroelectric field E_pyr_, which occurs when the crystal temperature changes [33] and is associated with the cooling of the droplet surface due to water evaporation and heating of the crystallization front [34].

**Figure 3:**
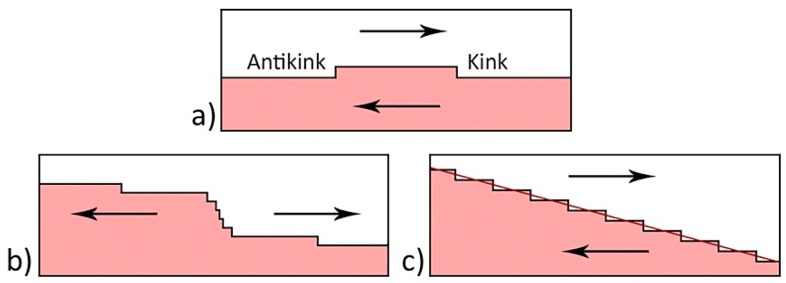
Schematic images of domain walls: (**a**) The formation of elementary steps, (**b**) macrostep with increased kink concentration, and (**c**) domain walls with uniform kink distribution. The arrows indicate the spontaneous polarization directions.

During polarization reversal, the elementary steps (pairs of kinks and antikinks) with a height of one lattice constant (Figure 3a) were generated at the domain wall, after which the charged kinks and antikinks moved along the polar axis [36]. The domain wall deviates from the polar direction and becomes inclined if the kinks are distributed uniformly (Figure 2a and Figure 3c). The nonuniform kink distribution along the domain wall led to the formation of macrosteps with an increased kink concentration, which can be observed by PFM (Figure 2b and Figure 3b). The wall motion velocity is proportional to the kink concentration, so the macrosteps seem to have moved along the neutral domain wall during polarization reversal.

**Figure 4:**
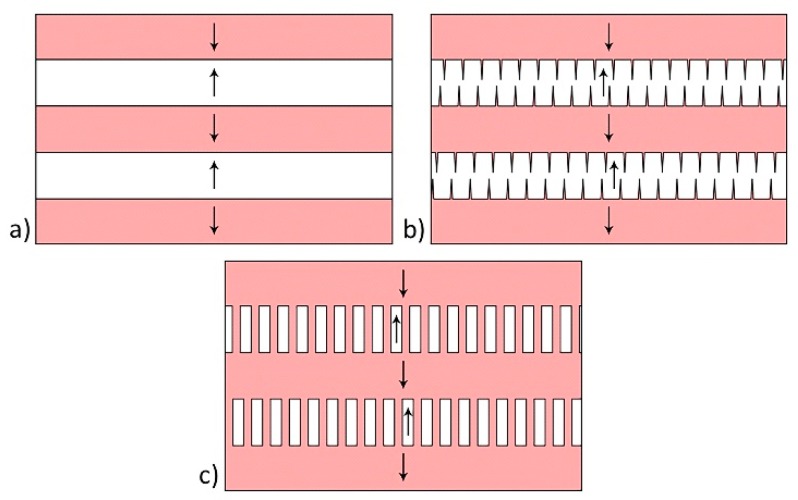
Schematic images of needle-like domain ensembles formation: (**a**) Initial domain structure, (**b**) polarization switching in one direction, and (**c**) merging of the projecting parts of charged domain walls. The arrows indicate the spontaneous polarization directions.

The formation of needle-like domain ensembles can be attributed to existence of an internal bias field E_b_, which impeded polarization switching in one direction (Figure 4). For E_pyr_ < E_b_, the initial stripe domains with flat charged walls (Figure 4a) switched in one direction, and the charged domain walls acquired a zigzag shape (Figure 4b). The merging of the projecting parts of the charged domain walls led to the formation of isolated needle-like domains (Figure 4c).

**Figure 5:**
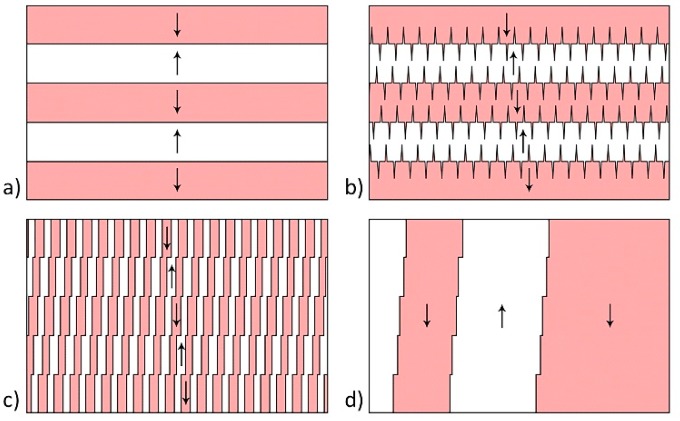
Schematic images of irregularly shaped domains formation: (**a**) Initial domain structure, (**b**) switching in both directions, (**c**) pass-through switching in stripe domains, and (**d**) long domains with neutral domain walls and charged steps. The arrows indicate the spontaneous polarization directions.

For E_pyr_ > E_b_ (Figure 5a) the strip domains switching in both directions (Figure 5b) led to a pass-through switching, which resulted in the formation of long domains with neutral domain walls and charged steps at the locations of the charged walls of the stripe domains (Figure 5c). The large domains were formed upon further merging of through domains (Figure 1c and Figure 5d).

**Figure 6:**
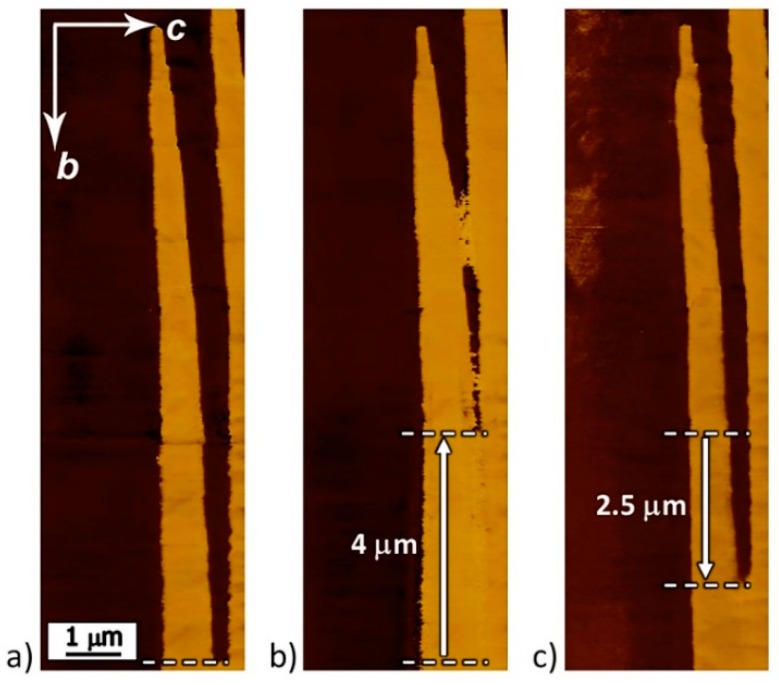
PFM images of domain evolution under the action of the pyroelectric field: (**a**) As-grown structure; (**b**) after heating from 10 °C to 35 °C with a heating rate of 120 °C/min, and (**c**) after cooling from 35 °C to 10 °C with cooling rate of 10 °C/min. Lateral PFM contrast. Arrows b and c show the directions of crystal axis.

The possibility of polarization reversal in the β-glycine crystals under the action of the pyroelectric field was experimentally validated. Figure 6 shows the change in the as-grown domain structure (Figure 6a) after heating (Figure 6b) and subsequent cooling (Figure 6c). After heating the crystal from 10 °C to 35 °C at a rate of 120 °C/min, the charged domain walls shifted by about 4 μm, and with subsequent cooling from 35 °C to 10 °C at a rate of 10 °C/min, they displaced in the opposite direction by about 2.5 μm.

**Figure 7:**
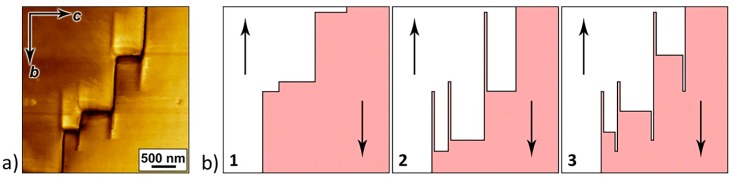
Nanodomains on charged domain walls. (**a**) Lateral PFM image of nanodomains on step-like domain walls. (**b**) Scheme of nanodomain formation as a result of cyclic motion of macrosteps: (**1**) Initial structure and displacements of charged domain walls (**2**) during heating and (**3**) during cooling. Arrows b and c show the directions of crystal axis. The arrows indicate the spontaneous polarization directions.

The nanodomains were sometimes detected at the step-shaped domain walls (Figure 7a). Within the proposed model, we explain the formation of such domains by the motion of macrosteps along the neutral domain walls under the action of the pyroelectric field caused by the temperature change (Figure 7b). When the sample with initial domain structure was heated, charged domain walls (Figure 7(b1)) were displaced by 1.5 μm, and residual nanodomains formed along the movement path (Figure 7(b2)). When the sample was cooled, the charged domain walls shifted in the opposite direction by 0.5 μm, and residual nanodomains with a different polarization direction appeared (Figure 7(b3)).

### 3.2. Local Polarization Reversal

**Figure 8:**
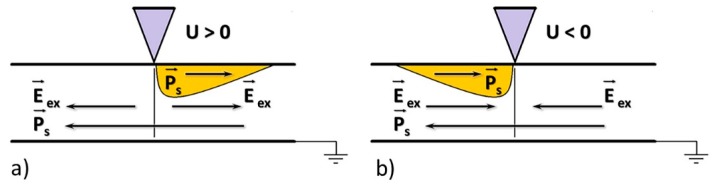
Schematic images of local polarization reversal. Local polarization reversal on a nonpolar cut by (**a**) positive and (**b**) negative voltage pulses applied to the probe.

The areas with stripe domains and irregularly shaped large domains were chosen for local polarization reversal. Two switching types were performed to identify the contribution of the backswitching to the domain formation: (1) With withdrawal of the probe immediately after the end of the pulse and (2) without withdrawal of the probe. The polarization reversal on a nonpolar cut occurred under the action of the in-plane component of the electric field created by the probe (Figure 8) [25]. The shallow domains (shown in yellow) were formed in the areas where the local electric field was above the threshold field, and the stable polarization state (opposite to initial polarization) appeared.

**Figure 9:**
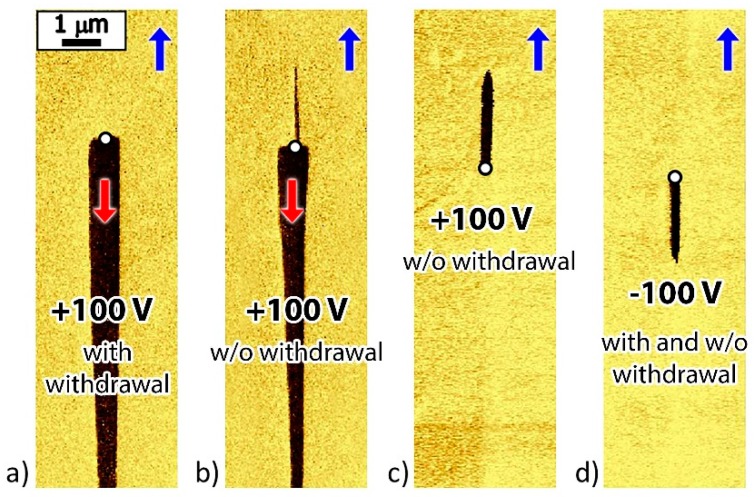
PFM images of the domains written by local polarization reversal: By a positive pulse (**a**) with probe withdrawal, (**b**,**c**) without probe withdrawal, and (**d**) negative pulse with and without probe withdrawal. Red and blue arrows indicate the spontaneous polarization directions.

After the local polarization reversal by a positive voltage pulse (U = 100 V; *t* = 15, 30, and 60 s) with the probe withdrawal, wedge-shaped domains with a length above 7 μm were formed (Figure 9a). At the same time, for β-glycine crystals, the polar field component created by the probe exceeds the threshold value only in a micron-size region (see Supplementary Materials).

After a local polarization reversal by negative voltage pulse (U = −100 V; *t* = 15, 30, and 60 s) with probe withdrawal, short (up to 2.5 μm) needle domains grew in the same direction as after the local polarization reversal by a positive pulse (Figure 9d). The observed switching unipolarity can be attributed to the presence of bias field E_b_, which prevents the switching by the external field. At the same time, the screening of the field localized under the probe led to the redistribution of the charge carriers. After the end of the voltage pulse, the charge carriers created a field of opposite sign, which resulted in the formation of reverse domains [24,37]. The origin of the internal bias field is unclear and could be attributed to intrinsic unipolarity of the glycine molecule.

Lack of probe withdrawal from the crystal surface facilitates backswitching due to the external screening of the depolarizing field by the current in the external circuit [21]. For local polarization reversal by positive voltage pulses, a partial or complete backswitching and the formation of reverse domains were observed (Figure 9b,c).

**Figure 10:**
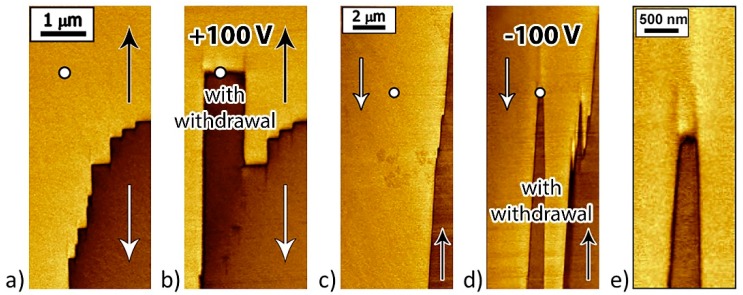
PFM images of written domains in the vicinity of as-grown domain wall with probe withdrawal. PFM domain images: (**a,c**) Before and (**b,d**) after local polarization reversal by a voltage with (**b**) positive and (**d**) negative pulses. (**e**) Zigzag-shaped head-to-head charged domain wall formed by local polarization reversal. The arrows indicate the spontaneous polarization directions.

After the local polarization reversal by a positive pulse (U = 100 V; *t* = 15, 30, and 60 s) with probe withdrawal, a flat charged tail-to-tail wall was formed at the probe location due to the displacement of the existing domain wall (Figure 10a,b).

For the local polarization reversal by a negative pulse (U = −100 V; *t* = 30 s) with probe withdrawal, a zigzag-shaped (head-to-head) wall (Figure 10e) formed at the probe location (Figure 10c,d). Switching in the areas up to 2 µm from the probe can be attributed to the field produced by injected charges [38,39,40].

**Figure 11:**
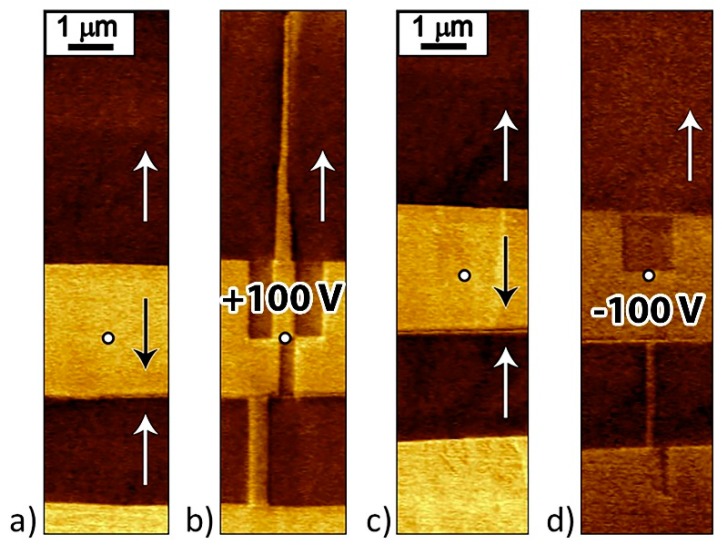
PFM images of written domains in the vicinity of as-grown domain wall without probe withdrawal. Domain structure (**a**,**c**) before and (**b**,**d**) after local polarization reversal by (**b**) positive and (**d**) negative voltage pulses. The arrows indicate the spontaneous polarization directions.

After the local polarization reversal by a pulse of positive U > 0 and negative voltage U < 0 (U = ±100 V; *t* = 30 s) without probe withdrawal partial or full backswitching and formation of reverse domains were observed (Figure 11).

**Figure 12:**
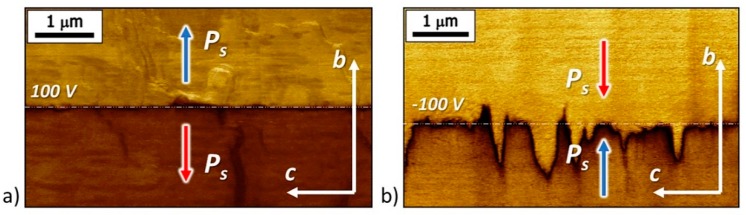
PFM images of the charged domain walls: (**a**) Tail-to-tail and (**b**) head-to-head. Domain walls were written by applying of (**a**) positive (100 V) and (**b**) negative (−100 V) voltage pulses with duration of 60 s at points located along the straight dotted line with period 1.5 μm.

The two types of charged domain walls written by the probe differed in morphology. The tail-to-tail walls were straight and smooth, while the head-to-head walls were jagged and zigzag-shaped (Figure 12). To quantify the smoothness of the walls, we measured the roughness value for both wall types in the 6 μm-length sections perpendicular to the polar axis. For the tail-to-tail walls, the roughness R_a_ was 15 nm, and about 200 nm for the head-to-head walls. This also confirms the earlier assumption that the charged tail-to-tail walls are conductive, which accelerates the bulk screening of the depolarizing fields created by the bound charges, and that the head-to-head walls are not conductive, which leads to a change in the shape of the domain wall to reduce the density of bound charges.

**Figure 13:**
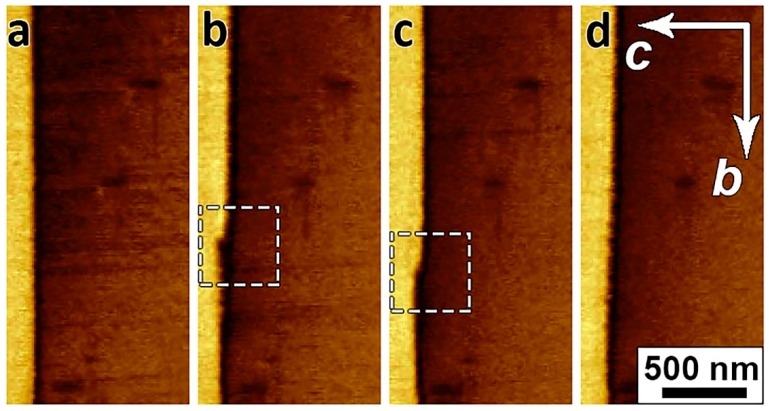
PFM images of the macrostep motion along the neutral domain wall: (**a**) 0, (**b**) 10, (**c**) 30, and (**d**) 50 min since the start of measurement. Arrows b and c show the direction of crystal axis.

A significant backswitching effect was demonstrated: The domains obtained by local polarization reversal slowly became short and narrow. It is shown that the backswitching was carried out by the motion of macrosteps along the neutral domain walls (Figure 13). Backswitching can be partial or even complete—until the domain’s disappearance.

## 4. Conclusions

A detailed study of the as-grown domain structure in β-glycine allowed us to identify three domain types with charged domain walls, the formation of which is attributed to the influence of growth layers and pyroelectric fields. The formation of nanodomains, observed in β-glycine for the first time, is attributed to the cyclic motion of the charged macrosteps caused by the influence of the alternating pyroelectric field that appeared during the heating–cooling cycle. The performed measurements showed that the domain structure changes with slight heating and/or cooling. The observed significant unipolarity of the local polarization reversal in β-glycine was attributed to the existence of an internal bias field. The effects of spontaneous backswitching after external field removal, and “anomalous” growth of the domains with polarization direction that did not coincide with the direction of the applied field, were detected. For the first time in organic ferroelectrics, we show that the formation in-field domains grown on nonpolar cuts, as well as their reduction after the field removal occur by the motion of kinks in the polar direction and the formation of macrosteps. It was shown that as-grown domain structure and its change under electric field in β-glycine are qualitatively similar to those found in uniaxial inorganic ferroelectrics. The control of the domain structure by an external local electric field provided by the PFM tip was demonstrated.

## Supplementary Materials

The following are available online at https://www.mdpi.com/1996-1944/12/8/1223/s1, Figure S1: The distribution of the electric field under the probe in a β-glycine single crystal; Figure S2: AFM images of crystal surface and corresponding PFM images of as-grown domain structures.
