# Supplementary material for: Domain Diversity and Polarization Switching in Amino Acid β-Glycine
Source: Materials (Basel). 2019 Apr 15;12(8):1223. doi: 10.3390/ma12081223 (PMC6514944; doi:10.3390/ma12081223)
Supplement: Supplementary file 1 [file materials-12-01223-s001.pdf]

materials

Article

Domain Diversity and Polarization Switching in Amino Acid β-Glycine

Daria Vasileva [1], Semen Vasilev [1,2], Andrei L. Kholkin [1,3,*] and Vladimir Ya. Shur [1]

1 School of Natural Sciences and Mathematics, Ural Federal University, Ekaterinburg 620000, Russia; daria.vasileva@urfu.ru (D.V.); vasilev.semen@gmail.com (S.V.); vladimir.shur@urfu.ru (V.Y.S.)
2 Department of Chemical Science, Bernal Institute, University of Limerick, V94 T9PX Limerick, Ireland
3 Department of Physics & CICECO-Aveiro Institute of Materials, University of Aveiro, 3810-193 Aveiro, Portugal
* Correspondence: kholkin@ua.pt; Tel.: +351-234-247-025

Supplementary Materials

The distribution of the electric field in the sample was calculated by the equation 1 [1]:

$$E = \frac{1}{2\pi\varepsilon_0}\frac{C_{tip}V}{\varepsilon+1}\frac{z}{(z^2+y^2+(x-R)^2)^{3/2}}, \quad (1)$$

where $\varepsilon_0 = 8.85 \cdot 10^{-12}$ F/m, $\varepsilon = 4.5$, $R = 35 \cdot 10^{-9}$ m, V = 100 V, x, y, z – coordinates.

The capacitance of the probe-sample system was calculated by the equation 2 [2]:

$$C_{tip} = 4\pi\varepsilon_0 R \frac{1+\varepsilon}{1-\varepsilon} log\left(\frac{2}{1+\varepsilon}\right) \quad (2)$$

where R is the effective tip radius.

According to the distribution of the electric field under the probe in β-glycine crystal (Fig. S1), it decreases by an order of magnitude at the distance about 150 nm from the probe contact and is negligible at the long distances (above 500 nm). However, the field created by the charged kinks may lead to further domain growth in the area with zero external electric field. This effect has previously observed on non-polar cuts of lithium niobate single crystals [3].

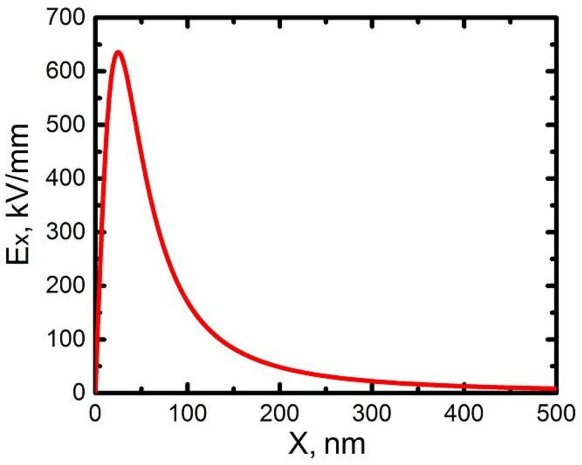

Figure S1. The spatial distribution of the electric field produced by the probe in β-glycine single crystal. The X-axis is oriented along the polar *b* axis.

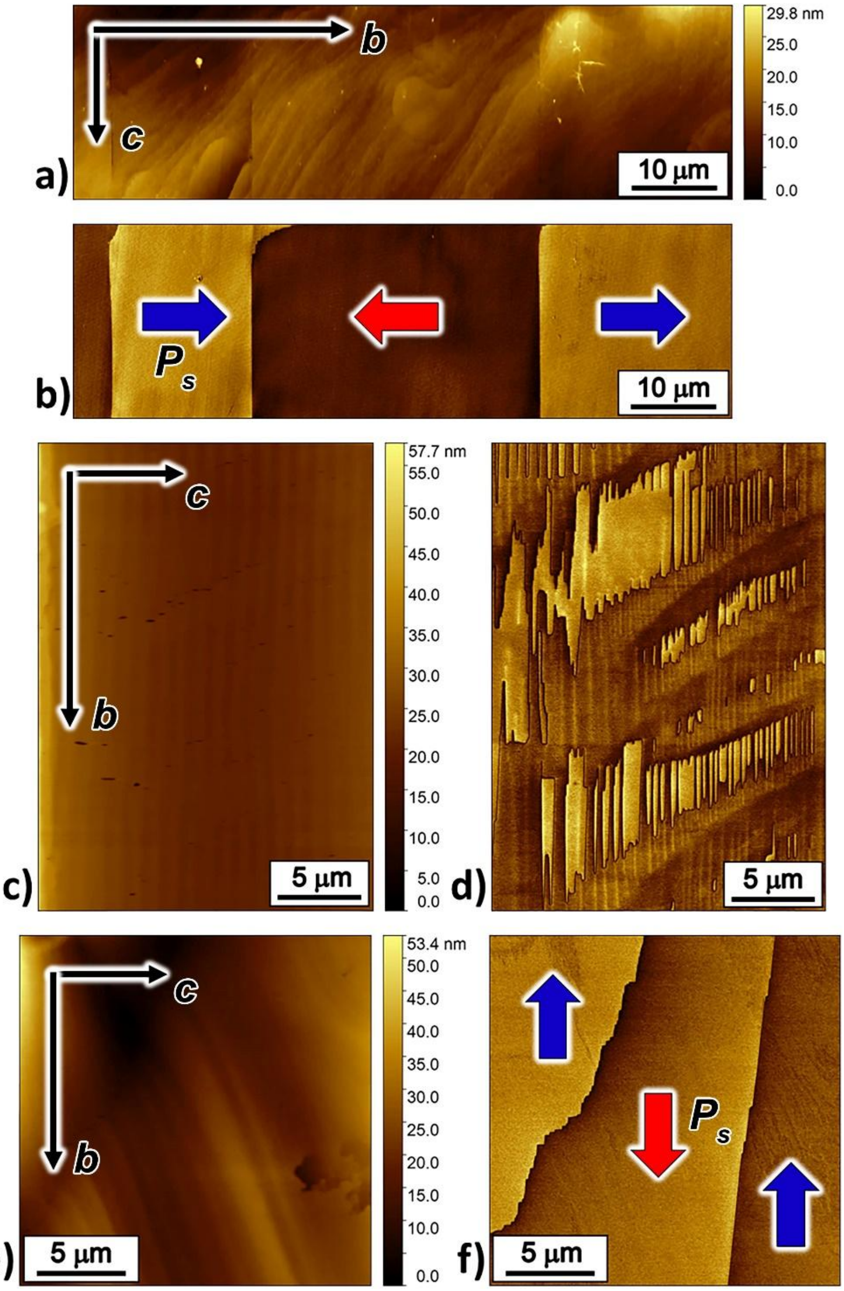

Figure S2. (**a, c, e**) AFM topography of crystal surface and (**b, d, f**) corresponding PFM images of as-grown domain structures. Lateral PFM contrast on non-polar surfaces of β-glycine representing three types of as-grown domain structures: (**b**) strip-like domains with flat charged domain walls, (**d**) quasiperiodic ensembles of needle-like domains, (**f**) large domains with irregular shaped domain walls. Red and blue arrows indicate the direction of spontaneous polarization. Arrows *b* and *c* show the directions of crystallographic axes.

References

1. Mele, E. J. Screening of a point charge by an anisotropic medium: Anamorphoses in the method of images. *Am. J. Phys.* **2001**, *69*, 557–562. Available online: https://aapt.scitation.org/doi/10.1119/1.1341252 (accessed on 12.03.2019).
2. Kalinin, S.V.; Bonnell, D.A. Imaging mechanism of piezoresponse force microscopy of ferroelectric surfaces. *Phys. Rev. B.* **2002**, *65*, 125408. Available online: https://journals.aps.org/prb/abstract/10.1103/PhysRevB.65.125408 (accessed on 12.03.2019).

3. Alikin, D.O.; Ievlev, A.V.; Turygin, A.P.; Lobov, A.I.; Kalinin, S.V.; Shur, V.Ya. Tip-induced domain growth on the non-polar cuts of lithium niobate single-crystals. *Applied Physics Letters* **2015**, *106*, 182902. Available online: aip.scitation.org/doi/pdf/10.1063/1.4919872?class=pdf (accessed on 12.03.2019).

© 2019 by the authors. Licensee MDPI, Basel, Switzerland. This article is an open access article distributed under the terms and conditions of the Creative Commons Attribution (CC BY) license (http://creativecommons.org/licenses/by/4.0/).
